# Supplementary material for: A Human and Rhesus Macaque Interferon-Stimulated Gene Screen Shows That Over-Expression of ARHGEF3/XPLN Inhibits Replication of Hepatitis C Virus and Other Flavivirids
Source: Viruses. 2022 Jul 28;14(8):1655. doi: 10.3390/v14081655 (PMC9414520; doi:10.3390/v14081655)
Supplement: Supplementary file 1 [file viruses-14-01655-s001.zip › supplementary figures.pdf]

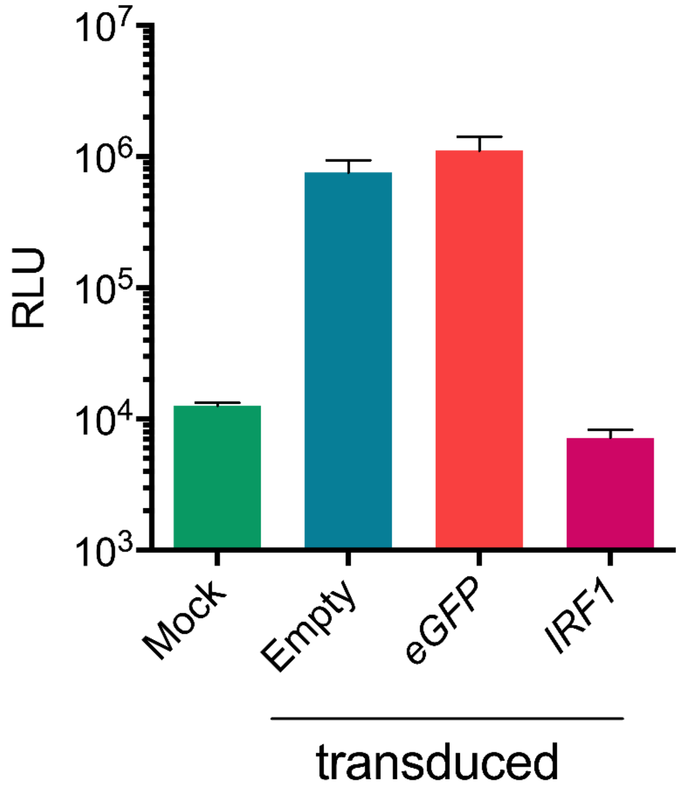

Figure S1. **<u>Optimisation of the ISG screen using HCV-SGR RNA.</u>** WT HCV-SGR RNA was transfected into cells previously transduced to express ISGs, including antiviral human *IRF1* gene. RLU was measured at 72 hpt compared to controls (empty vector, eGFP and mock transduced). All cell cultures had been treated with puromycin and therefore ‘mock’ denotes background RLU values in the assay. Values from triplicate wells were calculated and variation is shown as standard error of the mean.

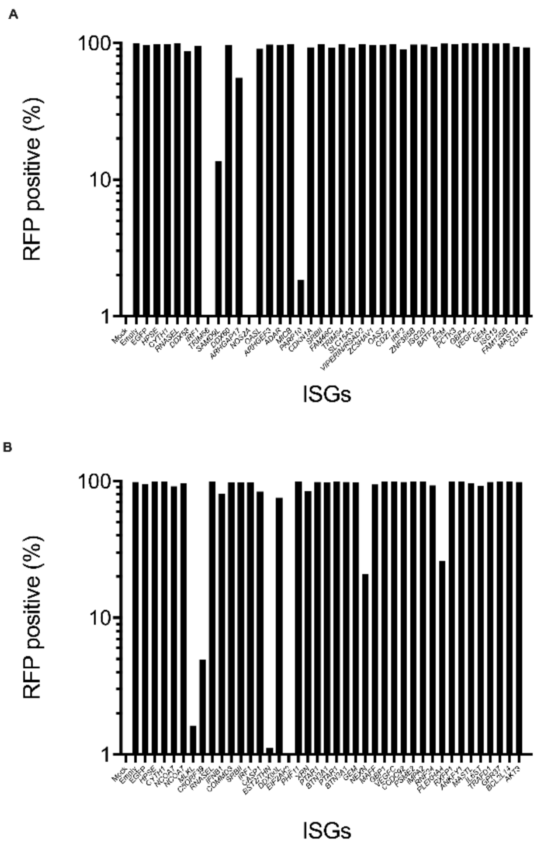

Figure S2. <u>Transduction efficiency of lentiviruses expressing ISGs.</u> In parallel assays from those shown in Figure 2, transduced cells were not selected using puromycin but instead were subjected to flow cytometry analysis to determine the abundance of transduced cells expressing

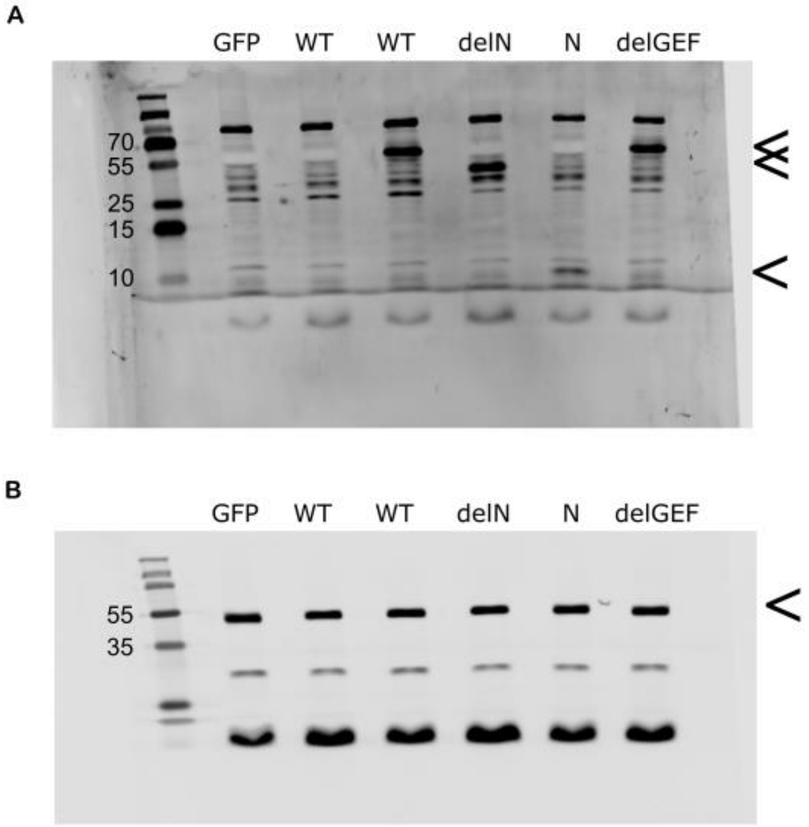

Figure S3. **<u>Expression of WT and mutant MYC-tagged ARHGEF3/XPLN.</u>** Representative immunoblots demonstrating production of MYC-tagged *ARHGEF3/XPLN* variants (**A**) and beta-tubulin loading control (**B**) in stably transduced Huh7 cells. Localisation of relevant bands is indicated with an arrow for WT/delGEF, delN, and N in (A), as well as beta-tubulin (in B). Molecular weights (kDa) of key proteins in the ladder are indicated on each gel.

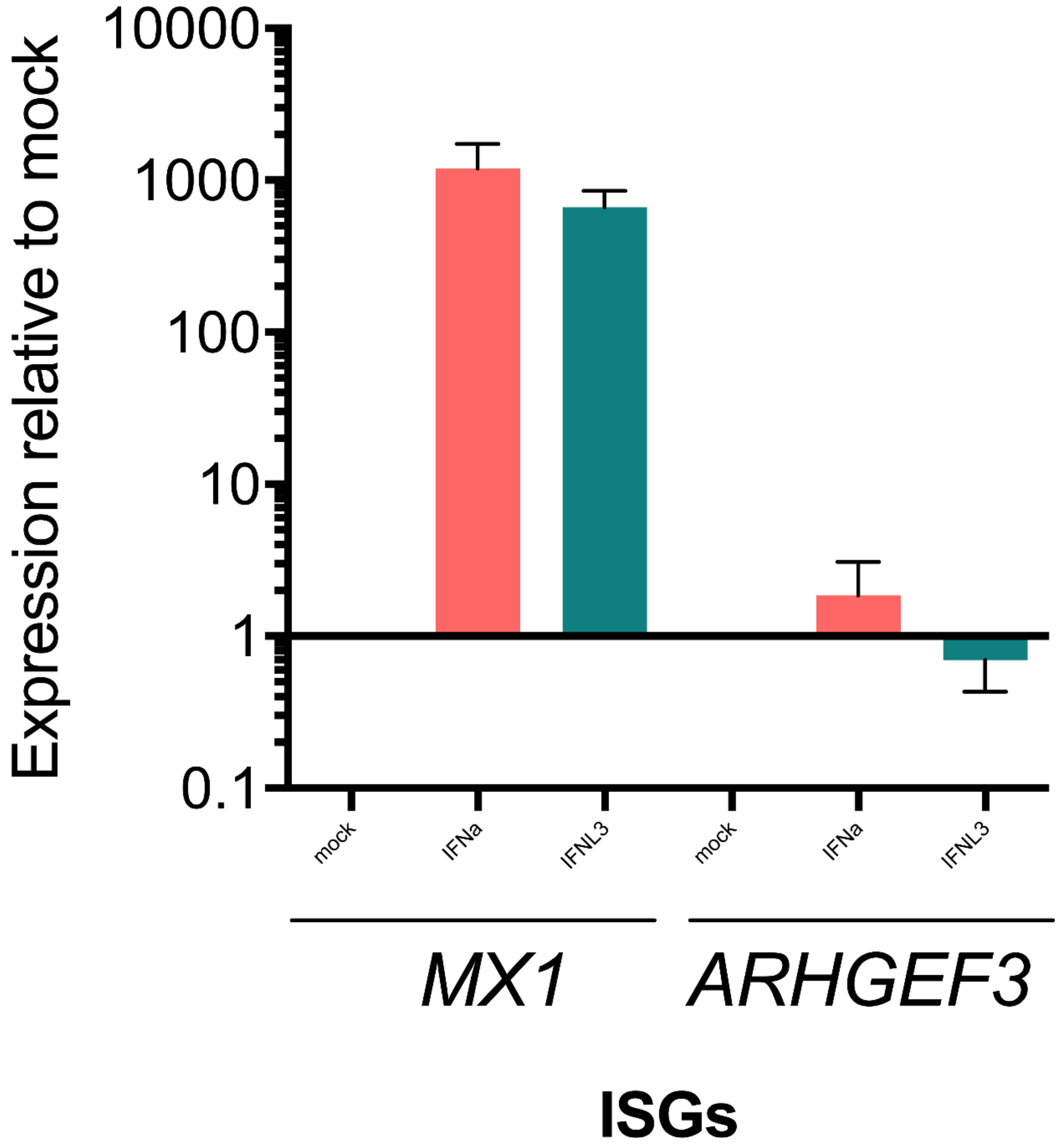

Figure S4. Expression of ARHGEF3/XPLN in Huh7 cells following IFN stimulation. Huh7 cells were stimulated with human recombinant IFN-alpha or IFN Lambda3 (1000IU/mL and 100ng/mL, respectively) for 24 hours and the levels of *MX1* and *ARHGEF3* (IFNa: pink; IFNL3: green) were determined. Changes in expression of *MX1* and *ARHGEF3* are shown as fold-change compared to mock stimulation. Values from duplicate wells were used and variation is shown as standard error of the mean.

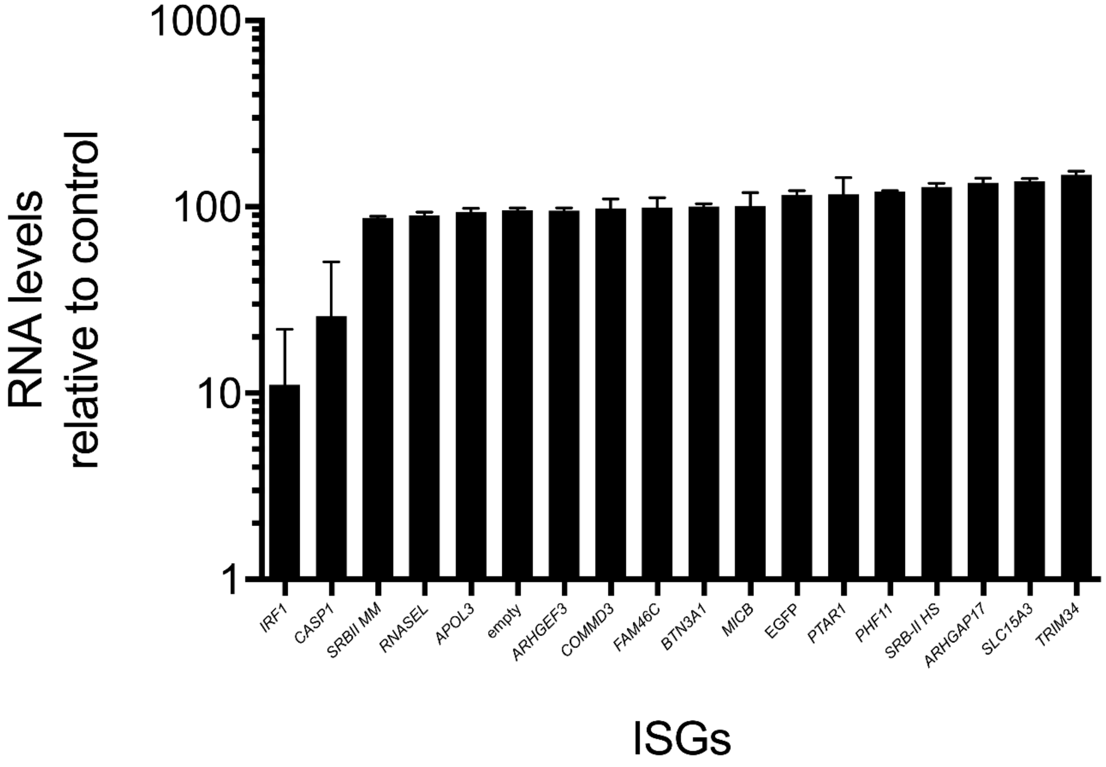

Figure S5. Antiviral activity of ISGs against stable HCV-SGR Huh7 cell lines. Relative RNA abundance (% of empty/EGFP controls) following puromycin selection. Huh7 cells were firstly transduced with lentiviruses expressing each of the ISGs for 48 hours and were then selected with puromycin for a further 48 hours.
